# Performance review of regional emergency medical service pre‐arrival cardiopulmonary resuscitation with or without dispatcher instruction: a population‐based observational study

**DOI:** 10.1002/ams2.273

**Published:** 2017-04-02

**Authors:** Hidetada Fukushima, Yasuyuki Kawai, Hideki Asai, Tadahiko Seki, Kazunobu Norimoto, Yasuyuki Urisono, Kazuo Okuchi

**Affiliations:** ^1^ Department of Emergency and Critical Care Medicine Nara Medical University Kashihara City Nara Japan; ^2^ Department of Emergency Nara Prefectural General Hospital Nara City Nara Japan; ^3^ Department of Emergency Yodogawa Christian Hospital Osaka Japan

**Keywords:** Cardiopulmonary arrest, emergency medical service, pre‐hospital care/medical control

## Abstract

**Background:**

To investigate variations in emergency medical service (EMS) pre‐arrival cardiopulmonary resuscitation (CPR), including both bystander CPR without dispatch assistance and dispatch‐assisted CPR (DACPR).

**Methods:**

We carried out an observational study by implementing EMS pre‐arrival CPR reports in three fire agencies. We included adult, non‐traumatic, and non‐EMS witnessed out‐of‐hospital cardiac arrests. This reporting system comprised the dispatch instruction process and bystander CPR quality based on evaluations by EMS crews who arrived on the scene. Bystander CPR was categorized as “ongoing CPR” if the bystander was performing CPR when the EMS reached the patient's side and “good‐quality CPR” if the CPR was performed proficiently. We compared the frequencies of ongoing and good‐quality CPR in the bystander CPR already started without dispatch assistance (CPR in progress) group and DACPR group.

**Results:**

Of 688 out‐of‐hospital cardiac arrests, CPR was already started in 150 cases (CPR in progress group). Dispatcher CPR instruction was provided in 368 cases. Among these, callers started chest compressions in 162 cases (DACPR group). Ongoing CPR was performed in 220 cases and was more frequent in the DACPR group (128/162 [79.0%] versus 92/150 [61.3%], *P* < 0.001). Good‐quality CPR was more frequent in the CPR in progress group, but the difference was not statistically significant (36/92 [39.1%] versus 42/128 [29.0%], *P* = 0.888).

**Conclusions:**

Ongoing CPR and good‐quality CPR were not frequent in EMS pre‐arrival CPR. Detailed analysis of dispatch instructions and bystander CPR can contribute to improvement in EMS pre‐arrival CPR.

## Background

For victims of sudden cardiac arrest (CA), bystander cardiopulmonary resuscitation (CPR) is a critical intervention for survival to hospital discharge.^1^, ^2^ The frequency of bystander CPR, however, still remains low, ranging from approximately 30% to 40%.^2^, ^3^ The reasons for bystanders not performing CPR are panic, lack of knowledge, being insufficiently confident to perform CPR, or the fear of unintentionally harming the patient.^4^, ^5^ Emergency medical service (EMS) dispatchers, who take emergency calls, can encourage and assist untrained or less confident rescuers to identify CA and start chest compression before EMS arrival.^6^, ^7^ It is reported that EMS dispatcher instruction for CPR can double the bystander CPR rate.^8^ Rea *et al*.^9^ reported that both dispatch‐assisted CPR (DACPR) and bystander CPR without dispatcher assistance are associated with improved survival for CA patients when compared to no bystander CPR. However, dispatcher instruction for CPR and bystander CPR are more complex than simple yes/no categorical variables. Both dispatchers and bystanders show wide variations in performance which can affect outcomes.^10^, ^11^ To improve EMS pre‐arrival CPR, these performances should be evaluated. Performance data, however, are limited in pre‐hospital settings and are not available from the widely used Utstein template database.^12^ To investigate and characterize the performance of both bystander CPR which was already started at the time dispatchers took calls (CPR in progress) and DACPR, we implemented an EMS pre‐arrival CPR reporting system in regional fire departments.

## Methods

This study was approved by the ethical committee of Nara Medical University (Kashihara City, Japan).

### Study settings

We undertook a population‐based, prospective observational study of EMS pre‐arrival CPR in three local Japanese fire departments (Nara, Chuwa, and Yamato‐Koriyama), which cover an area of 552.5 km^2^ with 680,131 residents, from 1 November, 2013 to 31 March, 2015. In this study, we included adult, non‐traumatic, and non‐EMS witnessed CA. Cases were also excluded if events occurred at medical facilities or had do not resuscitate orders.

To identify CA, dispatchers follow the pre‐defined protocol in Nara. If a bystander has already started CPR, EMS dispatchers tell the bystander to continue the procedure until EMS arrival. If CPR has not been started, dispatchers ask emergency callers whether the patient is responsive or not. If the patient is not responsive, they then ask whether the victim is breathing normally. Depending on the breathing descriptions obtained, such as “breathing abnormally”, “snoring weakly” or “not breathing”, EMS dispatchers consider the possibility of CA.^13^ Once CA is suspected, the dispatchers instruct the caller to unlock the door if appropriate, so that the EMS crew can immediately gain access to the patient. The dispatcher then provides the caller with detailed instructions for chest compression or conventional cardiopulmonary resuscitation with rescue breaths. For untrained callers, dispatchers provide compression‐only CPR in cases with presumed cardiac causes. If the callers cannot move the victims onto a hard flat surface, dispatchers instruct callers to leave the victims in situ and start CPR instruction. Once CPR is started, dispatchers instruct callers to continue the procedure until EMS arrival. In accordance with the policy to keep telephone lines open for other emergency calls in each agency, dispatchers are not required to hold CA calls until EMS arrival.

### Emergency medical service pre‐arrival CPR report form

**Figure 1:**
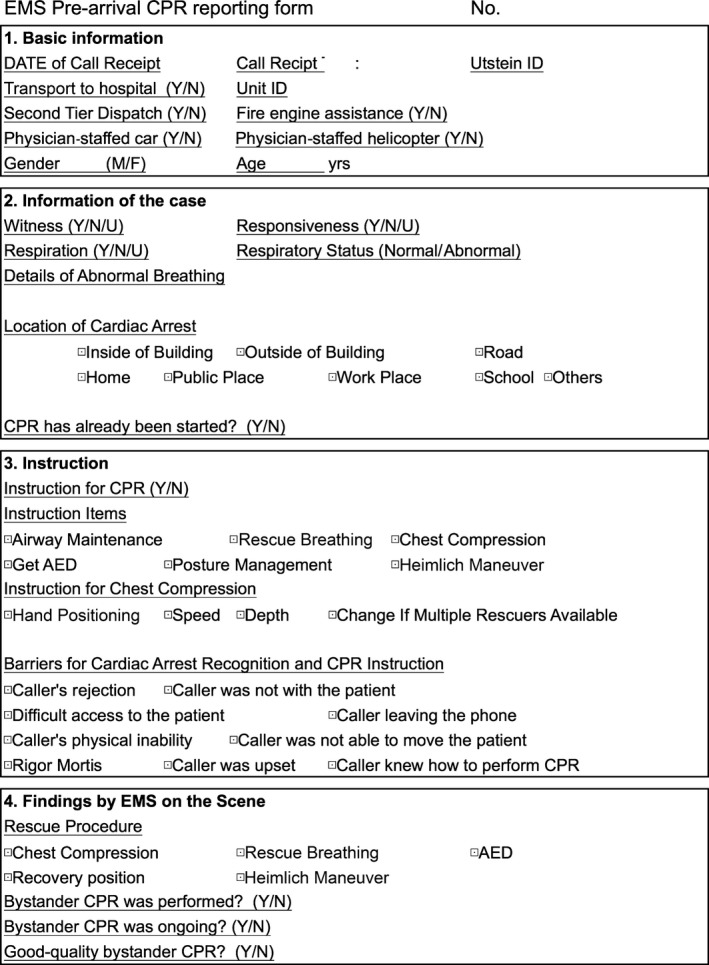
Emergency medical service (EMS) pre‐arrival cardiopulmonary resuscitation (CPR) reporting form used in this study to investigate bystander CPR with and without EMS dispatch assistance. AED, automated external defibrillator; N, no; U, unsure; Y, yes.

We implemented a reporting system of EMS pre‐arrival CPR in the three fire departments (Fig. 1). In accordance with agency personal information policy, the review of dispatch audio recordings by investigators was not included in this study. Dispatchers were required to record basic case information (including patient age, gender, witness status, and location of CA), dispatcher recognition of CPR need (yes/no), provision of CPR instruction (yes/no), and barriers to CA identification and CPR instruction. Dispatchers chose barriers described on the sheet when they encountered obstacles to provision of CPR instruction. These barriers were: the caller rejected CPR instruction, the caller was emotionally distressed, the caller left or hung up the phone, the caller was not with the patient, the caller had difficulty accessing the patient, the caller had physical limitations preventing CPR, the caller was unable to move the patient to a hard, flat surface, the caller knew how to perform CPR, and rigor mortis.^14^, ^15^, ^16^

In this reporting system, bystander CPR without chest compression is not regarded as CPR. The EMS personnel categorized bystander CPR as “ongoing CPR” if the bystander was performing CPR when the EMS crew arrived at the patient's side. Additionally, if EMS crews recognized ongoing CPR, they reported whether the compressions were properly performed in terms of compression rate (at least 100/min), depth (at least 2 inches or 5 cm), and appropriate hand position. If CPR performance meets all these criteria, they report the CPR as “good‐quality CPR”. Chest compression on patients not lying on a hard surface was considered poor chest compressions.

### Statistics

We compared patient profiles between CPR in progress and DACPR groups. We further characterized CPR performance among the two groups with respect to continuity and effectiveness. Data were presented as medians and interquartile ranges for continuous variables and numerically with percentages for categorical variables. Continuous variables were compared using the Mann–Whitney *U*‐test. The χ^2^‐test was used to assess associations between categorical variables. All statistical analyses were two‐sided and carried out using computer software (spss version 22; SPSS, Chicago, IL, USA). Results were considered to be statistically significant at a *P* value <0.05.

## Results

**Figure 2:**
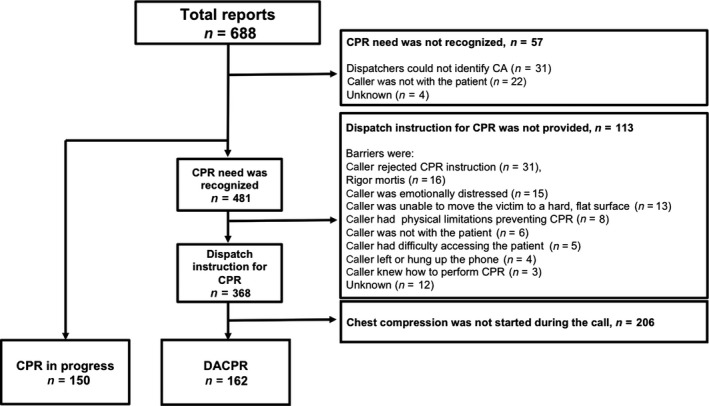
Overview of this study to investigate the performance of bystander cardiopulmonary resuscitation (CPR) in cases of cardiac arrest (CA) before arrival of the emergency medical service. DACPR, dispatch‐assisted cardiopulmonary resuscitation.

We obtained reports of 688 calls in total during the survey period. Among these, CPR need was not recognized in 57 cases and bystander CPR had already been started before EMS calls in 150 cases (CPR in progress). Out of 481 cases in which dispatchers recognized CPR need, CPR instructions could not be provided in 113 cases due to: the caller rejected CPR instruction (*n* = 31), rigor mortis (*n* = 16), the caller was emotionally distressed (*n* = 15), the caller was unable to move the patient to a hard, flat surface (*n* = 13), the caller had physical limitations preventing CPR (*n* = 8), the caller was not with the patient (*n* = 6), the caller had difficulty accessing the patient (*n* = 5), the caller left or hung up the phone (*n* = 4), the caller knew how to perform CPR (*n* = 3), and unknown reasons (*n* = 12). Dispatchers started CPR instruction in 368 CA cases. Among these, callers started chest compressions during calls in 162 cases (DACPR). In the remaining 206 cases, callers did not start chest compressions during calls (Fig. 2). The characteristics of the study group are described in Table 1.

**Table 1:** Characteristics of out‐of‐hospital cardiac arrest cases in which bystander cardiopulmonary resuscitation (CPR) was carried out with emergency medical service dispatch assistance (DACPR) or not (PCR in progress)

	Total (*n* = 312)	CPR in progress (*n* = 150)	DACPR (*n* = 162)	*P*‐values
Patient's gender				
Male, *n* (%)	175 (56.1)	80 (53.3)	95 (58.6)	0.345
Patient's age, years, median (IQR)	79 (66.8–86.0)	79 (65.0–86.0)	79 (69.0–87.0)	0.590
Location				
Private residence, *n* (%)	287 (92.0)	138 (92.0)	149 (92.0)	0.994
Witness, *n* (%)	98 (31.4)	53 (35.3)	45 (27.8)	0.148
Bystander				
Family member, *n* (%)	254 (81.4)	119 (79.3)	135 (83.3)	0.245

All continuous variables are presented as median and interquartile range (IQR); categorical variables are expressed as numbers and percentages.

The majority of patients were men (175/312 [56.1%]) with a median age of 79 years (interquartile range, 66.8–86.0 years) and events occurred mostly in private residences (287/312 [92.0%]). Cases were witnessed in 98/312 (31.4%) and the majority of bystanders were family members (254/312 [81.4%]; Table 1). Characteristics were similar between the two groups.

Emergency medical service crews arriving at the scene recognized 92/150 (61.3%) cases of ongoing CPR in the CPR in progress group. In the DACPR group, the frequency of ongoing CPR was significantly higher at 128/162 (79.0%) (*P* = 0.001). Out of 220 cases of ongoing CPR, EMS crews identified 83 as good‐quality CPR. The frequency of good‐quality CPR was higher in the CPR in progress group, but the difference was not statistically significant when compared to the DACPR group (Table 2; 36/92 cases [39.1%] versus 47/128 cases [29.0%], *P* = 0.888).

**Table 2:** Frequency of ongoing and properly performed bystander cardiopulmonary resuscitation (CPR), grouped according to commencement with emergency medical service dispatch assistance (DACPR) or not (CPR in progress)

	CPR in progress (*n* = 150)	DACPR (*n* = 162)	*P*‐value
Ongoing CPR, *n* (%)	92 (61.3)	128 (79.0)	0.001
Good quality CPR, *n* (%)	36 (39.1)	47 (29.0)	0.888

All variables are expressed as number (%).

## Discussion

Using this EMS pre‐arrival reporting template, we stratified bystander CPR based on dispatcher assistance, continuity, and quality. The frequency of ongoing CPR was significantly higher in the DACPR group than in the CPR in progress group. However, the numbers of good‐quality CPR were disappointing in both bystander CPR groups.

In this study, bystanders already started CPR in 150 cases at the time dispatchers took calls. Several studies have indicated that trained lay rescuers are more likely to perform bystander CPR than untrained laypersons.^4^, ^17^ We did not investigate the training status of rescuers in this study, but we presumed that many bystanders who had already started CPR were rescuers with training experience. However, both ongoing and good‐quality CPR were not frequent in this group. It is reported that CPR skills deteriorate soon after training,^18^ and the quality of layperson CPR is assumed to be poor. Very few studies have investigated the quality of layperson‐performed CPR because EMS pre‐arrival CPR data is notoriously difficult to collect. However, these studies showed that layperson CPR rarely achieves guideline parameters, with the CPR fraction reported to be especially low.^19^, ^20^ Although some rescuers perform good CPR, others perform CPR with insufficient knowledge and may stop chest compressions on their own judgement. According to the protocol in the study areas, dispatchers instruct rescuers to continue the procedures if they had already started bystander CPR and are not required to provide detailed CPR instruction. Our results indicate it is likely that dispatchers failed to assist those bystanders in continuing chest compressions. Even with callers who already started CPR, dispatchers can still improve the quality of bystander CPR by assessing how they are performing and assisting them in performing correct chest compression until the EMS arrives.

Dispatchers recognized 481 CA cases by phone, but could not provide CPR instructions in 113 cases. Dispatch‐assisted CPR is not a straightforward procedure and there can be many obstacles that prevent CPR instruction. Studies that reviewed the performance of DACPR have revealed that a certain proportion of CA cases were not eligible for CPR instruction because of callers’ refusal or physical factors.^15^, ^16^ In this current study, these were the major barriers to CPR instruction.

Dispatchers provided CPR instructions in 368 cases and DACPR was successfully started during the calls in 162 cases (44.0%). The success of DACPR in getting bystander CPR started has not been well investigated. Recent studies showed that success rates are reported to be 40% and 50%.^15^, ^16^ Although the success rate of DACPR was less than 50% in this study, dispatcher instructions for CPR was effective for ongoing CPR (128/162 [79.0%]). However, the quality of chest compression assessed by EMS at the scene was poor and dispatch instructions did not have a positive effect on the quality of CPR. The frequency of good‐quality CPR was less than 30% in this group (47/128 cases [29.0%]). Cardiopulmonary resuscitation simulation studies showed that the quality of chest compressions by laypersons is generally suboptimal. Studies using simplified dispatch instruction for CPR, coaching participants to “push as hard as you can” resulted in deeper compressions, but the depth of such compressions was also suboptimal.^21^ However, it is possible that continuous coaching for CPR by dispatchers help bystanders perform CPR properly. Continuous coaching for CPR quality using audio or visual real‐time feedback systems is reported to be effective for high quality CPR.^22^ One simulation study showed that even elderly laypersons aged 50–75 can perform chest compression with acceptable quality for 10 min with continuous coaching by phone.^23^ The effect of coaching on CPR quality during the DACPR process is yet to be clarified, but continuous coaching by dispatchers can be a surrogate for real‐time feedback system for bystander CPR with optimal quality. By providing continuous CPR coaching for bystanders who started CPR regardless of DACPR instruction, dispatchers can contribute to more frequent high quality CPR provision in prehospital settings.

This study showed that the majority of dispatch CPR instructions failed to provide DACPR and the quality of bystander CPR was poor. Our results indicate that there is room for dispatchers to contribute to improvement of high quality EMS pre‐arrival CPR. Strategies for effective CPR instructions for cases where CPR is in progress and where bystanders have not started CPR should be considered, based on detailed evaluations of bystander CPR during the EMS pre‐arrival period.

There are substantial limitations to be considered in this study. The first and most important limitation was a lack of a recording review process by research investigators, due to the personal information policy in the agency. Evaluation of the interaction between callers and dispatchers through review of audio recordings would have yielded considerable information regarding unidentified CA cases and incomplete dispatch instruction for CPR. Second, the quality of CPR was only evaluated at the scene, but not during the whole EMS pre‐arrival period. It is almost impossible to know how bystanders performed CPR through the whole EMS pre‐arrival period. Third, we failed to link the study dataset to any EMS attempted CPR data, including the Utstein database, and we were not able to explore the final outcome of the study group. Finally, the sample size of the study was small. There is a need to review EMS pre‐arrival CPR in a relatively large cohort.

## Conclusion

Our reporting system of EMS pre‐arrival CPR showed wide variations in ongoing CPR and CPR quality. Successful DACPR was not frequent in the cases where dispatchers provided instructions, and ongoing CPR or good‐quality CPR were also infrequent in EMS pre‐arrival CPR. Detailed analysis of dispatcher instructions and bystander CPR can contribute to improvement in EMS pre‐arrival CPR quality.

## Conflict of interest

None declared.
